# A comparative study of long interspersed element-1 protein immunoreactivity in cutaneous malignancies

**DOI:** 10.1186/s12885-020-07050-6

**Published:** 2020-06-17

**Authors:** Mohammad Ali Zolfaghari, Abbas Karimi, Elham Kalantari, Alireza Korourian, Alireza Ghanadan, Kambiz Kamyab, Nafiseh Esmaili, Amir Nader Emami Razavi, Zahra Madjd

**Affiliations:** 1 Department of Molecular Medicine, Faculty of Advanced Medical Sciences, Tabriz University of Medical Sciences, Tabriz, Iran; 2 Department of Dermatology, Razi Hospital, Tehran University of Medical Sciences, Tehran, Iran; 3 Oncopathology Research Center, Iran University of Medical Sciences, Tehran, Iran; 4 Department of Dermatopathology, Razi Dermatology Hospital, Tehran University of Medical Sciences, Tehran, Iran; 5 Autoimmune Bullous Diseases Research Center, Tehran University of Medical Sciences, Tehran, Iran; 6 Iran National Tumor Bank, Cancer Institute, Tehran University of Medical Sciences, Tehran, Iran

**Keywords:** Skin neoplasms, Retroelements, LINE-1 ORF1p, Immunohistochemistry, Tissue microarray, Biomarker

## Abstract

**Background:**

Skin cancer is the most common cancer worldwide and commonly classified into malignant melanoma (MM) and Nonmelanoma skin cancers (NMSCs), which mainly include basal cell carcinoma (BCC) and squamous cell carcinoma (SCC). The extent to which Long Interspersed Element-1 (LINE-1, L1) ORF1p is expressed in cutaneous malignancies remains to be evaluated. This study aimed to assess LINE-1 ORF1p immunoreactivity in various skin cancer subtypes.

**Method:**

The expression level of LINE-1 ORF1p was evaluated in 95 skin cancer specimens comprising 36 (37.9%) BCC, 28 (29.5%) SCC, and 31 (32.6%) melanoma using the tissue microarray (TMA) technique. Then the association between expression of LINE-1 encoded protein and clinicopathological parameters was analyzed.

**Results:**

We showed that LINE-1 ORF1p expression level was substantially higher in BCC and SCC patients compared with melanoma samples (*p* < 0.001). BCC cases had a higher LINE-1 histochemical score (H-score) compared with SCC cases (*p* = 0.004). In SCC samples, a lower level of LINE-1 ORF1p expression was associated with age younger than the mean (*p* = 0.041). At the same time, no significant correlation was found between LINE-1 ORF1p expression and other clinicopathological parameters (all *p* > 0.05).

**Conclusions:**

According to our observation, LINE-1 ORF1p immunoreactivity in various skin tumor subtypes extends previous studies of LINE-1 expression in different cancers. LINE-1ORF1p overexpression in NMSCs compared with MM can be considered with caution as a tumor-specific antigen for NMSCs.

## Background

Cutaneous malignancy is one of the most common tumors involving millions of humans around the world and, unfortunately, on the rise. Skin cancers are generally classified as malignant melanoma (MM), which represents only 4% of skin cancer cases and non-melanoma skin cancers (NMSC). NMSC includes two major subtypes of BCC and SCC, amongst others [1, 2]. The incidence rate of NMSC is 18–20 times higher than that MM; however, it constitutes a relatively small percentage of skin cancer deaths [3]. BCC and SCC are rarely fatal, whereas 65–74% of deaths due to cutaneous cancer are caused by malignant melanoma [4]. The high cure rate is associated with BCC and SCC, especially when the lesion is small and diagnosed in early stages [1, 5]. The early-stage melanoma may be hard to detect but is curable, whereas metastatic form has a poor prognosis and low survival rate [6].

Several risk factors including individual fair skin, blond hair/red hair, freckling, age, gender, personal or family histories, exposure to environmental UVR, high levels of arsenic in drinking water, polycyclic aromatic hydrocarbons, smoking, genetic syndromes and taking immunosuppression are known to induce cutaneous malignancies [7, 8]. The development of skin cancer takes place in a multi-step process by which the accumulation of mutations that can result in genomic instability, which is a prominent feature of most cancers, such as melanoma [9]. One of the mechanisms that are associated with genomic instability is the activation of Transposable Elements (TEs).

TEs are categorized into two subgroups of DNA transposons and RNA transposons or retrotransposons [10]. Retrotransposons can propagate themselves through the human genome using RNA mediators. Long Interspersed Element-1 (LINE-1, L1) accounts for about 17% of the human genome; however, their ability to construct eukaryotic genome structure is a key factor throughout the evolution [11]. Most of these elements are 5′-truncated and incapable of retrotransposition, but intact and full-length L1 elements are still potent and active sequences in the human genome [12]. A full-length L1 element is ∼6 kbp in length and divided into three parts including 1) a 5′ untranslated region (UTR) comprises an internal RNA polymerase II promoter; 2) two open reading frames (ORF1 and ORF2); 3) a 3′ UTR which is finished with a variable polyA tail. ORF1 and ORF2 are translated into an RNA-binding protein (40-kDa) that has chaperone activity and a protein with endonuclease and reverse-transcriptase activities (150- kDa), respectively [10]. ORF1p trimers have a prominent role than ORF2p in retrotransposition events such that ORF1p is generated more than the ORF2p [13, 14]. The function of these elements is intrinsically silenced in their promoters by epigenetic modification and several trans-acting factors [14, 15].

In normal somatic cells, methylation is a powerful mechanism of control over the activation of the retrotransposable elements to avoid genomic instability, chromosomal defects, and other genomic rearrangements [16]. Global DNA hypomethylation is a critical feature of human cancers; it may be due to the transcriptional inactivation of LINE-1 elements [17]. LINE-1 promoter hypomethylation has been described in various tumors, including lung cancer, colorectal cancers, breast cancer, prostate cancer, liver cancer, ovarian cancer, and esophageal cancer [14].

In non-small cell lung cancer (NSCLC), transcriptional activation of L1 promoters by hypomethylation results in genomic instability and unfavorable prognosis [18, 19], and in colorectal cancer emerges as an early specific marker [20]. Both invasive and in situ lesions of breast cancer have shown incomplete methylation of LINE-1 promoter resulting in reduced overall survival rate and treatment-resistant in younger patients [21, 22]. In a recent study, LINE-1 hypomethylation levels have been observed in melanoma tumors thicker > 4 mm compared with normal melanocyte primary cell cultures [23].

It has revealed that the production of ORF1p due to the LINE-1 expression in in vitro transfected cells is 1000- to 10,000-fold higher levels compare to ORF2p [24]. More than half of human cancers express LINE-1 ORF1p so that it could be considered as a highly specified tumor marker [17]. Up to now, there is no data regarding the expression of LINE-1 ORF1p in various skin cancer subtypes. Based on previous studies [9, 25, 26], it seems that MM may harbor a higher level of genomic instability and heterogeneity compared with BCC and SCC. This hypothesis raised the question as to whether LINE-1 ORF1p expression differs among various skin neoplasms, and if so, to what extent? In order to achieve immunohistochemically expression data of LINE-1 ORF1p, we aimed to study the LINE-1 ORF1p expression levels by tissue microarray (TMA) in skin cancer specimens comprising BCC, SCC, and melanoma.

## Methods

### Characteristics of patients and samples

A total of 139 formalin-fixed paraffin-embedded (FFPE) specimens from various skin cancer patients were included in the study. These archival tissue samples were obtained from patients with primary skin cancer diagnosed in the Razi and Imam Khomeini Hospitals of Tehran University of Medical Sciences, Tehran, Iran. Data are presented for patients diagnosed with cancer between 2013 2016. Medical records were reviewed to obtain – clinicopathological parameters comprising age, gender, lesion type, tumor size, ulceration, metastasis, tumor-infiltrating lymphocytes (TILs), perineural invasion (PNI), Clark and Breslow thickness scales (in melanoma), and histological grade (in SCC). Not only the patient’s data have no interference in their diagnosis and treatment, but they also were completely kept anonymous. The Research Ethics Committee of Tehran University of Medical Sciences has approved all the study procedures (Ref no: IR.TUMS.REC.1394.1733).

### TMA construction

The entire skin cancer TMA blocks were created as described previously [27–30]. Before TMA construction, all hematoxylin and eosin-stained slides were reviewed by our pathologist colleague (A-K). Then the most morphologically representative areas of tumors in FFPE blocks were annotated. To constructed TMA blocks, 0.6 mm diameter punches from the region of interests (already annotated) transferred into one empty recipient paraffin block. A TMA block was mad in triplicate 0.6-mm cores of the marked area of each sample using tissue-arraying equipment (MiniCore; ALPHELYS, Plaisir, France). Then 4 μm sections were cut from the completed recipient array blocks and transferred to adhesion microscope slides. These glass slides were used for immunohistochemically staining of LINE-1 ORF1p antigenicity.

### Immunohistochemistry

In brief, all the TMA slides were deparaffinized at 60 °C for 20 min to be allowed for dehydration with two different alcohol in grade. The solution of hydrogen peroxide 3% (v/v) for 20 min at room temperature was used for blocking endogenous peroxidase. Following washing the slides three times, the antigen retrieval step was performed. This step was followed by autoclaving for 10 min at 121 °C using 10 mM solution of sodium citrate (pH 6.0). Then slides were incubated with the primary monoclonal antibody (1:500 dilution), Anti-LINE 1 ORF1 (EMD Millipore, Cat. No MABC1152, CA, USA) overnight at 4 °C. The slides were then incubated with a secondary antibody cocktail of anti-rabbit/antimouse Envision (Dako, Denmark) for 30 min. Finally, substrate and chromogen (3,3-diaminobenzidine DAB; Dako) was added to the slides and followed by counterstaining with hematoxylin visualize antigen (Dako, Denmark). After dehydration in graded alcohols, slides were cleared in xylene (Dako) and mounted for examination.

As mentioned, colorectal samples are positive for LINE-1 ORF1p expression. We used them as a positive control for confirming the immunolabeling of Anti-LINE 1 ORF1. We also used normal skin samples as negative control. Then we optimized and validated antibody titer and dilution on CRC samples as positive control and ten normal skin samples as a negative control. To simultaneously compare the immunoreactivity of the sample, all experiments were run with the same experimental set-up.

### Immunostaining assessment

Immunostaining of LINE-1 ORF1p was independently reviewed by two well-experienced academic pathologists (AK and AG) who were blinded to the patients’ outcome and other clinical findings. A consensus outcome was reached in case of discordance. A semi-quantitative scoring approach was used to analyze the staining intensity in slides [30]. First, slides were evaluated at 10× magnification to find positive cores and overall distribution of the tumor cells. Then the percentage of the stained area was examined in high-power fields. The staining intensity was scored as 0 (negative), 1 (weak), 2 (moderate), and 3 (strong). The percentage of positive tumoral cells was scored as < 25%, 25–50%, 50–75, and > 75% of tumor cells. The histochemical score (*H-score*) was calculated for each case by multiplying the staining intensity and the percentage of positive tumor cells, which yielded a range from 0 to 300. In this study, the mean *H-score* was chosen to categorize samples as with high or low LINE-1 ORF1p expression.

### Statistical analysis

For comparison of LINE-1 staining scores in various skin cancer subtypes, we did a two-sided Student’s t-test to understand the difference between each group means. Moreover, Pearson’s chi-square and Pearson’s R tests were used to analyzing the significance of association and correlation between LINE-1 ORF1p expression and clinicopathological parameters. *P* values less than 0.05 were considered statistically significant.

## Results

Following tissue processing and immunohistochemistry staining of skin tumors, samples with missing one or two cores were excluded from the study. A total of 95 samples were included in this study. Of 95 cases, 28 (29.5%) were SCC, 36 (37.9%) BCC, and 31 (32.6%) MM. The present study comprises 67 and 28 male & female, respectively. There was a male predominance in 3 groups and male to female ratio in SCC, BCC, and MM were as follows: SCC (23 males and five females: 4.6), BCC (26 males and ten females: 2.6) MM (18 males and 13 females: 1.38). The mean age of patients in BCC, SCC, and MM subtypes of skin cancers were calculated as 70.44 ± 10.2, 67.23 ± 12.6, and 65.1 ± 14.2 years, respectively. Seven (19.4%), 2 (7.1%), and 6 (19.4) patients of BCC, SCC and MM subtypes of skin cancer had ulceration in pathological reports. In terms of invasive and in situ forms in SCC, 2 (7.14) patients had in situ, whereas 12 (42.8%) had invasive form, and for remaining of SCC patients, it was not available. Margin involvement was seen in 1 (2.8%), 2 (7.1%), and 7 (23.3%) patients of BCC, SCC, and MM patients. Tumor-infiltrating lymphocytes as a prognostic factor and PNI was found in 1 (3.6%) and 1 (3.6%) patients of SCC and 6 (19.4%) and 3 (9.7%) MM patients, respectively. Tumor size was available for 8 (28.6%) SCC cases with a mean value of 4-mm. Metastasis and local recurrence were available for 12 (38.7%) and 17 (54.8%) of MM patients, respectively. Moreover 7 (22.6%) MM patients had lymphovascular invasion. Melanoma lesions are categorized with Breslow thickness into ≤1 (thin melanoma) and > 1 mm (thick melanoma) [31]. Thin melanoma was found in 2 (6.5%) cases and thick melanoma in 8 (25.8%). In Clark’s system, melanomas are divided into two groups: group 1 (Clark levels I and II) and group 2 (Clark levels III through V). Seven (22.6%) melanoma cases were classified as group 2, and 6 (19.4%) as group 1 (Table 3), for the remaining it was not available. Tables 1, 2 and 3summarize the clinicopathological features of skin cancer subtypes.

**Table 1:** Association of expression LINE-1 ORF1p expression with clinicopathological parameters in BCC

Clinicopathological characteristics		Total Number (%)	ORF1p expression (mean ***H***-score = 170.4)		***P*** value
			High (> 170.4)	Low (< 170.4)	
Mean age ± SD (70.44 ± 10.2 years)	> 70	19 (52.8)	7 (36.8)	12 (63.2)	0.33
	≤70	17 (47.2)	9 (52.9)	8 (47.1)	
Gender	Male	26 (72.2)	10 (38.5)	16 (61.5)	0.24
	Female	10 (27.8)	6 (60)	4 (40)	
Ulceration	Yes	7 (19.4)	2 (28.6)	5 (71.4)	0.34
	No	29 (80.6)	14 (48.3)	15 (51.7)	
Histological subtypes	Nodular	19 (52.8)	10 (52.6)	9 (47.4)	0.19
	Pigmented	5 (13.9)	2 (40)	3 (60)	
	Infilterative	4 (11.1)	0	4 (100)	
	Adenoid	2 (5.6)	2 (100)	0	
	Micronodular	2 (5.6)	1 (50)	1 (50)	
	Metatypical	2 (5.6)	0	2 (100)	
	Sclerosing	1 (2.8)	0	1 (100)	
	Superficial	1 (2.8)	1 (100)	0	
Margin involvement	Yes	1 (2.8)	0	1 (100)	0.36
	No	35 (97.2)	16 (45.7)	19 (54.3)

**Table 2:** Association of LINE-1 ORF1p expression with clinicopathological parameters in SCC

Clinicopathological characteristics		Total Number (%)	ORF1P expression (mean ***H***-score = 111.84)		***P*** value
			High	Low	
Mean age + SD (67.23 ± 12.60 years)	> 67	11 (39.3)	6 (54.5)	5 (45.5)	**0.041**
	≤67	17 (60.7)	3 (17.6)	14 (82.4)	
Gender	Male	23 (82.1)	9 (39.1)	14 (60.9)	0.09
	Female	5 (17.9)	0	5 (100)	
Histological grade	Well	13 (46.4	2 (15.4)	11 (84.6)	0.2
	Moderate	11 (39.3)	5 (45.5)	6 (54.5)	
	Poor	4 (14.3)	2 (50)	2 (50)	
Tumor size	≤4 mm	4 (14.3)	2 (50)	2 (50)	0.1
	> 4 mm	4 (14.3)	0 (0)	4 (100)	
	No data	20 (71.4)	7 (35)	13 (65)	
Ulceration	Yes	2 (7.1)	0	2 (100)	0.31
	No	26 (92.8)	9 (34.6)	17 (65.5)	
Tumor-infiltrating lymphocytes	Yes	1 (3.6)	1 (100)	0	0.13
	No	27 (96.4)	8 (29.6)	19 (70.4)	
Perineural invasion (PNI)	Yes	1 (3.6)	0	1 (100)	0.48
	No	27 (96.4)	9 (33.3)	18 (66.7)	
Invasive and in situ forms	Invasive	12 (42.8)	7 (58.3)	5 (41.7)	0.25
	In situ	2 (7.14)	2 (100)	0	
	No data	14 (50)	0	14 (100)	
Margin involvement	Yes	2 (7.1)	0	2 (100)	0.31
	No	26 (92.8)	9 (34.6)	17 (65.4)

**Table 3:** Association of LINE-1ORF1p expression with clinicopathological parameters in MM

Clinicopathological characteristics		Total Number (%)	ORF1P expression (mean ***H-score*** = 46.27)		***P*** value
			High	Low	
Mean age ± SD (65.1 ± 14.2 years)	> 65	15 (48.4)	5 (33.3)	10 (66.7)	0.60
	≤65	16 (51.6)	4 (25)	12 (75)	
Gender	Male	18 (58.1)	6 (33.3)	12 (66.7)	0.53
	Female	13 (41.9)	3 (23.1)	10 (76.9)	
Ulceration	Yes	6 (19.4)	0	6 (100)	0.07
	No	18 (58.1)	7 (38.9)	11 (61.1)	
	No data	7 (22.6)	2 (28.6)	5 (71.4)	
Perineural invasion (PNI)	Yes	3 (9.7)	0	3 (100)	0.21
	No	20 (64.5)	7 (35)	13 (65)	
	No data	8 (25.8)	2 (25)	6 (75)	
Metastasis	Yes	12 (38.7)	6 (50)	6 (50)	0.26
	No	8 (25.8)	2 (25)	6 (75)	
	No data	11 (35.5)	1 (9.1)	10 (90.9)	
Local recurrence	Yes	17 (54.8)	7 (41.2)	10 (58.8)	0.16
	No	3 (9.7)	0	3 (100)	
	No data	11 (35.5)	2 (18.2)	9 (81.8)	
Tumor-infiltrating lymphocytes	Yes	6 (19.4)	0	6 (100)	0.19
	No	4 (12.9)	1 (25)	3 (75)	
	No data	21 (67.7)	8 (38.1)	13 (61.9)	
Lymphovascular invasion	Yes	7 (22.6)	1 (14.3)	6 (85.7)	0.79
	No	5 (16.1)	1 (20)	4 (80)	
	No data	19 (61.3)	7 (36.8)	12 (63.2)	
Margin involvement	Yes	7 (23.3)	2 (28.6)	5 (71.4)	0.5
	No	18 (60)	3 (16.7)	15 (83.3)	
	No data	6 (16.7)	4 (66.7)	2 (33.3)	
Breslow thickness	Thin (≤1 mm)	2 (6.5)	0	2 (100)	0.59
	Thick (> 1 mm)	8 (25.8)	1 (12.5)	7 (87.5)	
	No data	21 (67.7)	8 (38.1)	13 (61.9)	
Clark level	Group 1 (I, II)	7 (22.6)	1 (14.3)	6 (85.7)	0.33
	Group 2 (III-V)	6 (19.4)	0	6 (100)	
	No data	18 (58.1)	8 (44.4)	10 (55.6)	

### Analysis of LINE-1 ORF1p expression and its correlation with clinicopathological features

**Fig. 1:**
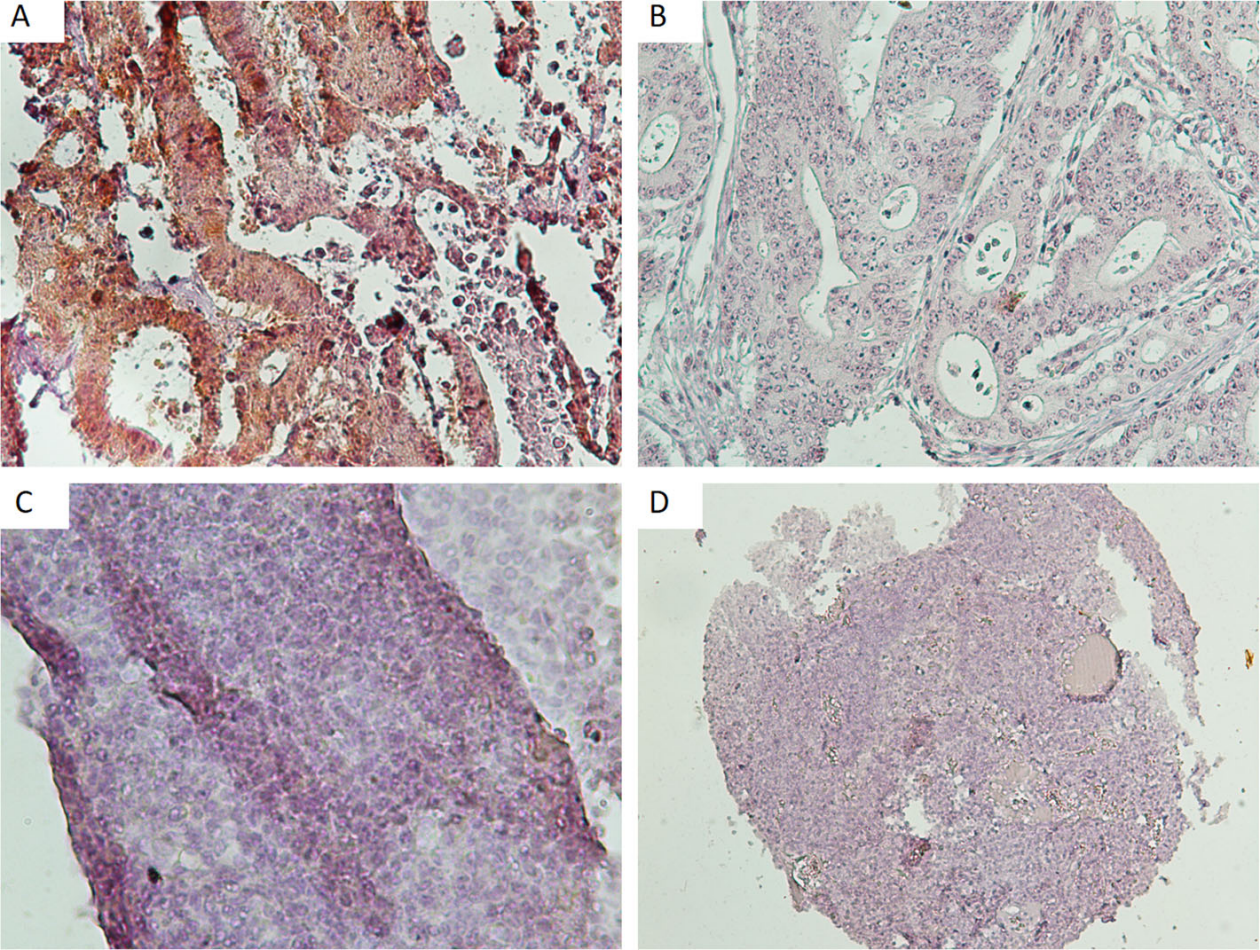
IHC staining of colorectal, surgical intestinal resection margins in colon cancer, and normal skin tissues. **a** Positive immunoreactive LINE-1 ORF1p in CRC, **b** negative surgical resection margin sample for LINE-1 ORF1p, **c** and **d** are representative for normal skin tissues with different magnifications

**Fig. 2:**
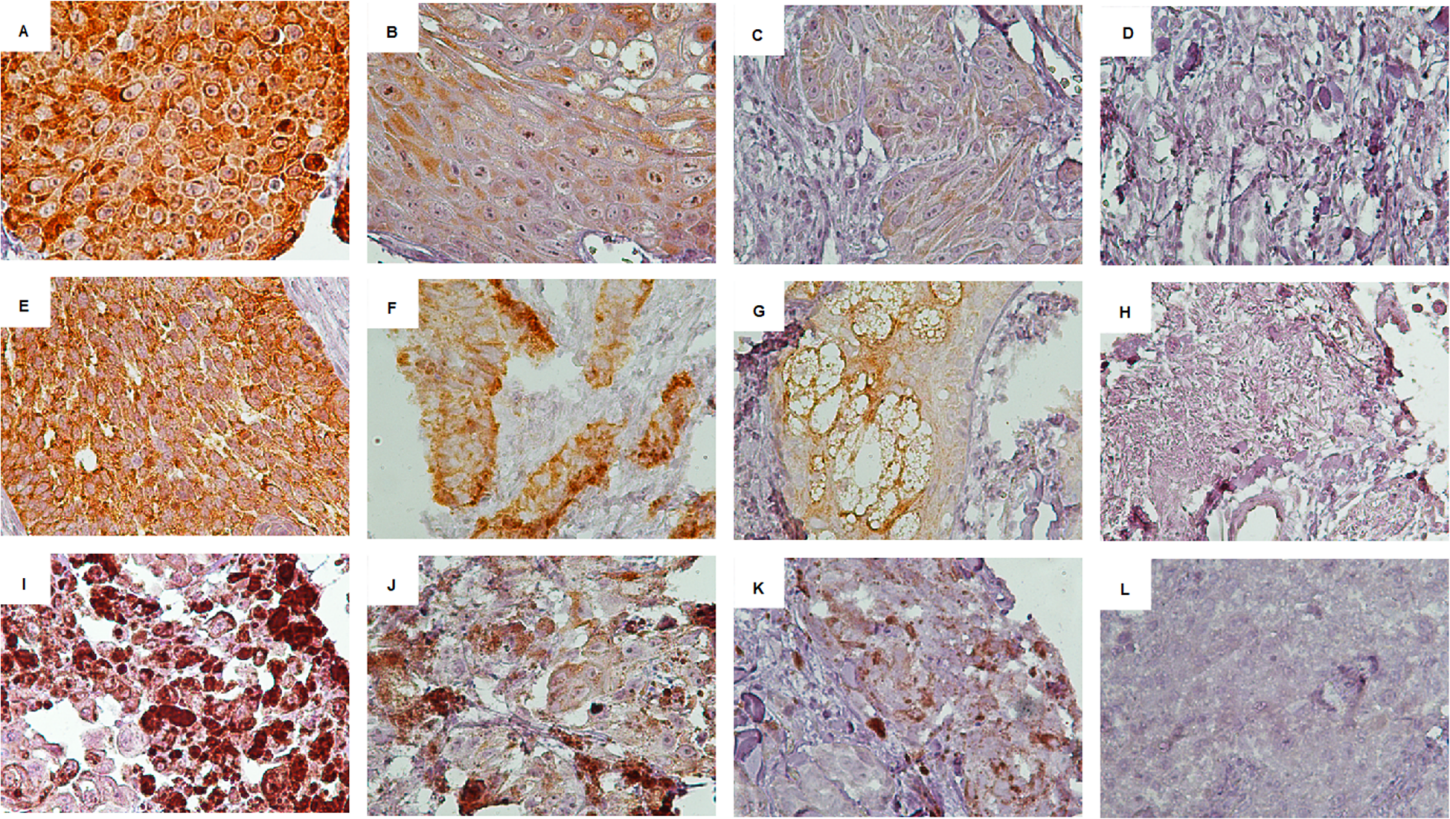
Immunohistochemical analysis of LINE-1ORF1p expression in different skin cancer subtypes. LINE-1 ORF1p expression in SCC: **a** + 3, strong; **b** + 2, moderate; **c** + 1, weak; **d** 0, no intensity. LINE-1ORF1p expression in BCC: **e** + 3, strong; **f** + 2, moderate; **g** + 1, weak; **h** 0, no intensity. LINE-1ORF1p expression in melanoma: **i** + 3, strong; **j** + 2, moderate; **k** + 1, weak; **l** 0, no intensity

**Fig. 3:**
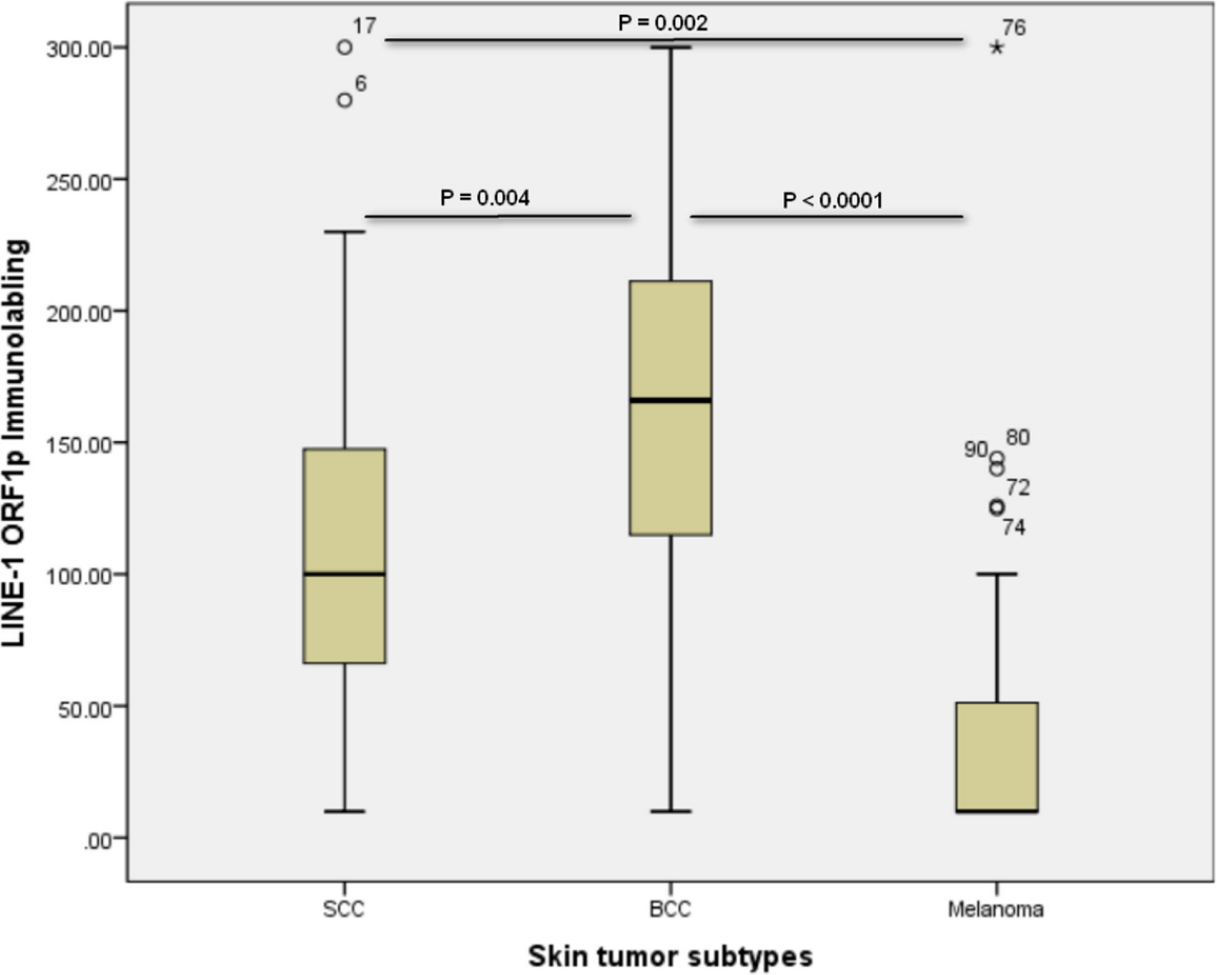
Box-plot diagram of LINE-1 ORF1p expression in skin tumor subtypes. In each panel, the vertical axis shows the total immunolabeling score (*H-score*)

We first tested LINE-1 ORF1p immunoreactivity on normal skin tissue and colorectal tissues (Fig. 1). Following optimization and validation of antibody titration and dilution on CRC and normal skin tissues as a positive and negative control, we did all controls and patients tests simultaneously. Each tumor type was divided into either lower (≤ mean of *H-score*) or higher (> mean of *H-score*) LINE-1 ORF1p expression. The mean *H-score* in BCC and SCC samples were 170.4 and 111.84, respectively, whereas the mean *H-score* in melanoma cases was 46.27. Twenty of 36 BCC samples (55.6%) expressed lower levels of LINE-1 ORF1p, while 16 (44.4%) cases expressed higher levels (Fig. 2). Low expression of LINE-1 ORF1p was seen in 19 (67.9%) of 28 SCC cases, while high expression was found in 9 (32.1%) samples (Fig. 2). Of the 31 melanoma samples, 22 (71%) had a low expression, and 9 (29%) showed a high expression of LINE-1 ORF1p (Fig. 2). We found a highly significant difference between mean *H-score* of LINE-1 ORF1p expression among the three tumor subtypes (all *p* < 0.01). Box plot diagram of LINE-1 ORF1p expression has been shown in (Fig. 3). Furthermore, the output of the Mann-Whitney *U* test revealed a significant difference in LINE-1 ORF1p expression between BCC and SCC (*p* = 0.004) and melanomas (*p* < 0.0001). Also, there was a significant difference between the expression of LINE-1 ORF1p in SCC and melanoma (*p* = 0.002). Whereas, we could not find a significant correlation between studied clinicopathological parameters and LINE-1 ORF1p expression in BCC and melanoma samples (all *p* > 0.05) (Tables 1 & 3). A trend was evident between LINE-1 ORF1p expression and ulceration (*p* = 0.07). In SCC samples, a lower level of LINE-1 ORF1p expression was associated with age lower than the mean (*p* = 0.041), while no significant correlation was found between LINE-1 ORF1p expression and other clinicopathological parameters (all *p* > 0.05) (Table 2). Regarding the different subtypes of lesions, we did not see any difference in LINE-1 ORF1p immunolabeling of superficial versus nodular BCC, which are two main histological types of BCC. Since the numbers are small and, therefore, would not support a chi-squared test for trend on a contingency with missing data. However, Fisher’s exact test also was not significant for this analysis (*P* > 0.99). We also could not find any positive trend between lentigo and superficial spreading melanoma both in statistical analysis, and pathological review. Also, it was the same for BCC superficial versus nodular.

## Discussion

In this study, we found LINE-1 ORF1p ORF1p immunoreactivity in various skin tumor subtypes that are in line with previous studies of LINE-1 expression in different cancers. As previously reported [32], ORF1p is expressed 200-fold than ORF2p, so tracking ORF1p throughout disease progression can provide valuable insights regarding retrotransposition events or the impact of LINE-1 expression on the genome.

Overall, LINE-1 ORF1p overexpression can be used as a specific hallmark for diagnosing human malignancies [17]. In the vast majority tumors including approximately 90% of breast, ovarian, pancreatic cancers, more than half of tubular gastrointestinal tract cancers comprising esophageal and colon cancers and also 50% of lung cancers and 40% of prostate tumors, ORF1p can be detected by immunohistochemistry [14, 17]. As mentioned, LINE-1 activation/expression is associated with genomic instability that is a hallmark and characteristics in malignant tumors, including melanoma. Because of this, we expected to get a wide range of LINE-1 expression in MM patients. In the current study, we found significant expression of ORF1p in BCC samples in comparison to SCC and melanoma. ORF1p immunolabeling in BCC samples was higher than those of melanoma samples in contrary to our hypothesis. The reason why the ORF1p expression in NMSC was higher than malignant melanoma requires more attention.

Carcinogenesis in skin tissues is associated with exposure to different environmental factors. Previous reports have shown that some environmental factors like gamma irradiation and X- rays some environmental agents such as benzo [a] pyrene (B [a]P), organochlorine pesticides, food-borne carcinogens, extremely low-frequency magnetic fields (ELF-MF) and some heavy metals like arsenic, aluminum increase L1 retrotransposition events [33–35]. Since the skin is the first line of defense for mentioned factors, so the higher expression of the level of ORF1 due to the exposure to agents is plausible.

Melanoma tumors have higher levels of genomic instability [9] and are associated with hypomethylation of genomic LINE-1 sequences [23, 36]. However, we expected to get more expression level of ORF1p in melanoma, what caused to observe such a decreased level in comparison to other subtypes remains a mystery for us. Likely, the monoclonal LINE-1 ORF1p antibody referenced in this study that recognizes the sequence corresponding to amino acids 35 to 44 of LINE-1 ORF1p (MENDFDELRE) has not the capability for targeting those sequences in melanoma. It seems that conventional antigen retrieval pathways are not sufficient for retrieving LINE1 ORF1p immunolabelling. In SCC cases, a trend towards increasing the correlation of ORF1p expression with age was observed, which supports the evidence that age can play a role in developing SCC.

We observed both cytoplasmic and nuclear pattern of LINE-1ORF1p expression in all of the samples, while the cytoplasmic pattern has been predominantly represented in some cancers [17]. In breast cancers, local relapse, as well as distal metastases and poorer overall survival with tumors displaying nuclear L1-ORF1p in contrast to cytoplasmic L1-ORF1p group, have been observed [37]. Distinguishing and quantification between cytoplasmic and nuclear expression can be highlighted in skin tumor subtypes in future studies.

Each genome harbors different copies of 80–100 potentially active L1 elements, and this partly explains the variability in somatic insertions of L1 elements within tumors [38]. How many full length - potentially active elements contribute to immunoreactivity for LINE-1 ORF1p and whether observed heterogeneity in these subsets of skin tumor subtypes is related to differences in the inherited complement of active L1 elements or not remains to be robustly evaluated. Evaluating such heterogeneity in melanoma cases may pave finding the reason for such differences. Since the tumor microenvironment is a key contributor in cutaneous malignancies progression, studying L1 hypomethylation of functional L1 promoters and their genomic sequences in precancerous lesions can offer a genuine reservoir for finding novel targets for both therapeutic purposes and risk assessments in skin cancers.

In this context and according to our findings, clinical information on the potential utility of LINE-1 ORF1p expression and activation as a novel biomarker in skin neoplasms are, however, limited, and future investigations should be directed towards identifying a correlation between LINE-1 expression and histopathological or diagnostic implication for practitioners is needed. If the value of ORF1p expression is clinically validated in SCC and BCC patients, such information will help clinicians make better decisions for prognosis and plan treatment and follow-up of patients.

## Conclusions

Evidence from basic and translational science indicates a correlation between LINE-1 induction and tumorigenesis, cancer progression, and therapeutic response. Despite ample evidence, the causal and mechanistic links between LINE-1ORF1p expression and the development of different cancers are still unraveled. According to our observation, LINE-1 ORF1p immunoreactivity in various skin tumor subtypes confirms previous studies of LINE-1 expression in different cancers. LINE-1 ORF1p overexpression in NMSCs compared with MM can be considered with caution as a tumor-specific antigen for NMSCs.

## Data Availability

The data that support the findings of this study are available from the corresponding author, [AK], upon reasonable request.

## Abbreviations

NMSCs: Nonmelanoma skin cancers
LINE-1, L1: Long Interspersed Element-1
ORF1p: Open reading frame 1 protein
MM: Malignant melanoma
BCC: Basal cell carcinoma
SCC: Squamous cell carcinoma
TMA: Tissue microarray
H-score: Histochemical score
UVR: Ultraviolet radiation
TEs: Transposable Elements
UTR: Untranslated region
ORF: Open reading frame
FFPE: Formalin-fixed paraffin-embedded
TILs: Tumor-infiltrating lymphocytes
PNI: Perineural invasion
IHC: Immunohistochemistry
